# Case of Santonine Poisoning

**Published:** 1896-06

**Authors:** 


### CASE OF SANTONINE POISONING.

(Translated by the late Dr. Samuel Lilienthal.)

A boy of eleven years took, from November to March 3.6 grammes Santonine and lots of worm-lozenges. He complained at first only of bellyache; during February he suffered from chronic cramps, with great restlessness and anguish. During March his legs became weaker, his voice hoarse, and finally aphonia set in. The boy sees fiery balls, lightning, and everything appears yellow. Face pale, pupils dilated and without reaction, severe vertigo and headache, nausea, vomiting, burning and pressure in the eyes; accelerated respiration, feels downcast, pulse 80; impossibility to walk, patellar reflex normal, sensibility preserved, constipation, and the catheter has to be used. He hears and understands everything, but cannot talk. During the pains long continued clonic twitchings in the legs, more than in the arms; contortions of the eyes, consciousness undisturbed. It took months before all symptoms disappeared, the last ones were debility and a disagreeable, copious sweating.

—*A. H. Z.*, July, 1890.

